# Identifying implementation strategies to increase the early detection of cardiac amyloidosis: a pre-implementation study

**DOI:** 10.3389/fcvm.2026.1810173

**Published:** 2026-07-22

**Authors:** Alexis M. Koskan, Jenice Guzman-Clark, Sherry L. Ball, Ralph Renger, Sandesh Dev

**Affiliations:** 1 College of Health Solutions, Arizona State University, Phoenix, AZ, United States; 2 Southern Arizona VA Health Care System, Tucson, AZ, United States; 3 Research Service Line, VA Northeast Ohio Healthcare System, Cleveland, OH, United States; 4 JUST Evaluation Services, Tucson, AZ, United States

**Keywords:** amyloidosis, cardiac amyloidosis (CA), early diagnosis, heart failure, implementation science, implementation strategies, transthyretin

## Abstract

**Introduction:**

Cardiac amyloidosis (CA) has been considered a rare disease. However, its prevalence is growing as healthcare practitioners have new, less invasive diagnostic tools that aid in the early detection of this disease. Bone scintigraphy with monoclonal protein testing is one such evidence-based, noninvasive screening option that can be offered in community healthcare facilities. This work aims to identify intervention strategies to improve the timely workup of transthyretin CA, including evidence-based practices such as bone scintigraphy and monoclonal protein testing.

**Methods:**

The team used rapid qualitative content analysis to develop a system map of care for cardiac amyloidosis, guided by both patient and healthcare expert perspectives. To explore the map's utility and accuracy, the team conducted follow-up in-depth interviews with clinicians. They explored clinicians' perspectives on strategies to facilitate the early diagnosis of CA and explored barriers and implementation strategies that can influence the diagnostic work-up for CA.

**Results:**

Clinicians provided recommendations for how to expand the map, particularly in terms of including additional healthcare professionals, screening strategies, and barriers to care for patients. After expanding the preliminary system map of care, the team identified 23 distinct strategies to improve the early detection of CA. Clinicians recommended additional strategies, such as patient and provider education, patient take-back, the use of artificial intelligence with electronic health record data, and the formation of healthcare teams specializing in cardiac amyloidosis, to enhance early detection and treatment of this condition.

**Discussion:**

This work can inform researchers and clinicians about selecting and implementing intervention and implementation strategies within health systems to enhance the early detection of cardiac amyloidosis.

## 1 Introduction

Advances in non-invasive diagnostics have led to rapid increases in diagnosing cardiac amyloidosis (CA), once considered a rare condition that, when undetected and untreated, leads to premature mortality, loss of physical function, and poor quality of life (1). These different CA conditions differ in the types of proteins that misfold, their prognosis, and the treatments they require (2). Further, ATTR-CA is more common than AL-CA (3), with an estimated US prevalence of 50,000–100,000 (4).

Patients with ATTR-CA often develop heart failure (HF), atrial fibrillation, and non-cardiac conditions such as neuropathy, spinal stenosis, and carpal tunnel syndrome (5). These symptoms are common and seemingly unrelated; therefore, clinicians may miss opportunities to diagnose CA. It is essential to describe both current and ideal clinical practices for the early diagnosis of ATTR-CA because awareness and communication of the constellation of early warning signs could save lives (6). However, despite ongoing advancements, including non-invasive diagnostic testing for CA (7), this systemic disease remains underdiagnosed and misdiagnosed, and is subject to diagnostic delays (8).

An evidence-based diagnostic work-up strategy, bone scintigraphy coupled with monoclonal protein testing, can identify patients with ATTR-CA or AL-CA. Bone scintigraphy, a nuclear medicine imaging technique (Tc-99 m PYP [pyrophosphate] or Tc-99m-HMDP [hydroxymethyl diphosphonate]), in combination with monoclonal protein testing, offers significant diagnostic opportunities, with a sensitivity of >99% and a specificity of 86% for detecting ATTR-CA (9). Patients with a characteristic scintigraphy scan and absence of monoclonal proteins do not require endomyocardial biopsy. Biopsy requires a heart tissue sample and is most often performed in advanced HF tertiary centers, whereas scintigraphy can be performed in all settings, including community healthcare facilities. As such, in 2022, the American College of Cardiology and the American Heart Association Heart Failure guidelines recommended that clinicians order bone scintigraphy and screen for light chains via monoclonal protein testing in patients with a clinical suspicion of CA (9). Despite this clear recommendation, suboptimal uptake of this workup strategy is likely, given healthcare providers' low clinical suspicion for ATTR-CA, which influences the tests, clinical work-up, and diagnoses patients receive (10).

Two research questions that guided this work include: (1) What are the barriers for patients to receive the work-up for the early diagnosis of CA (bone scintigraphy coupled with monoclonal protein testing)? and (2) What intervention strategies can lead to the evidence-based work-up for CA (bone scintigraphy coupled with monoclonal protein testing)? For this research, we utilized the first sets up the System Evaluation Theory (SET) to map the system of care and identify implementation strategies that lead to the timely workup for ATTR-CA (11). Another article describes the steps for utilizing SET and creating and updating the system map, including specific updates from the original draft to the final system map in greater detail (12). However, this exploratory study focuses on identifying implementation strategies and applying it to well-known implementation strategies for future implementation science research aimed at increasing the timely uptake of bone scintigraphy and monoclonal protein testing.

## 2 Materials and methods

We received Institutional Review Board approval from the Arizona State University Institutional Review Board (STUDY00017092). We first created a preliminary system map of care for the early detection of ATTR-CA that also serves as a standard operating procedure for the early diagnostic work-up for this condition. The map was informed by an expert panel of clinicians who treat CA patients and two CA patients. We conducted follow-up in-depth interviews with the clinicians to refine and update the map and to develop implementation strategies to increase uptake of the evidence-based approach to detecting CA using bone scintigraphy and monoclonal protein testing. Below, we describe these methods in more detail.

### 2.1 Expert panel participants

Using purposive sampling, the research team invited CA experts and patients attending a day-long CA symposium (a day-long CA symposium hosted on January 14, 2023) to participate in a 3-hour workshop panel on early detection of CA. CA experts in the panel represented multiple disciplines involved in the care of amyloidosis patients. The cardiologist who planned the CA symposium ensured the panel was diverse and contained the necessary expertise to provide input on the early detection of CA. An expert amyloid cardiologist identified two CA patients with different forms of the condition (e.g., one caused by ATTR and one caused by AL) to describe their experiences leading to the diagnosis of CA. See Table 1 for a description of the workshop attendees.

**Table 1:** Expertise of workshop and follow-up interview participants.

Participant type	Expert level	Workplace setting (for clinicians)	Participated in	
			Workshop	Follow-up interview
Patient with ATTR-CA	–	–	X	–
Patient with AL-CA	–	–	X	–
Hematologist	Local	Amyloidosis Center of Excellence (Hospital)	X	–
Primary care provider	Local	Community clinic	X	X
Cardiologist	International	Amyloidosis Center of Excellence (Hospital)	X	X
Cardiologist	National	Amyloidosis Center of Excellence (Hospital)	X	X
Cardiologist	Local	Community hospital	–	X
Cardiologist	Local	Amyloidosis Center of Excellence (Hospital)	–	X
Nuclear medicine	National	Academic medical center	X	X
Genetic counseling	Local	Amyloidosis Center of Excellence (Hospital)	X	X
Cardiac sonographer	Local	Community clinic	X	X
Neurologist	National	Amyloidosis Center of Excellence (Hospital)	X	X
Pathologist	National	Amyloidosis Center of Excellence (Hospital)	X	X

All participants completed written informed consent. The team conducted and recorded the panel discussion. A professional transcription company transcribed the panel discussion, and the research team conducted a matrix analysis to organize the results of this interdisciplinary audience of providers and patients (13). The team, comprised of clinicians, social science researchers, and a SET expert, met weekly to discuss the matrix analysis and to gain consensus on their coding. At this stage, the team did not seek information saturation, given that the goal was to map the various responses.

### 2.2 Follow-up interviews

Following the panel, the research team created a preliminary system map—a visual depiction of the steps leading up to bone scintigraphy and monoclonal protein testing—and recorded a video presentation of the map, which was emailed to all workshop clinician participants (See Table 1 for their expertise). This was also a method of member-checking, ensuring that the preliminary system map aligned with participants' shared perspectives. Patients were not interviewed at this stage of the research due to their lack of experience in healthcare delivery. The video presentation described the aim of refining the systems map and developing implementation strategies to increase the early detection of CA. Between April and June 2023, the research team invited participants to a web-based (Microsoft Teams) hour-long in-depth interview designed to refine the existing map and identify barriers and facilitators for implementation. (See Appendix 1 for the interview guide.) Two qualitative research experts (initials blinded for review) with doctorates in the health sciences took turns leading the interviews while the other took field notes. Additionally, team clinicians (initials) attended the interviews to ask follow-up questions or clarify clinical information. All follow-up interview participants completed written informed consent. The follow-up interviews lasted, on average, 62 min (range = 39.4–80.5 min). The team contacted one participant after her interview to clarify an implementation strategy that she proposed. Upon completing the in-depth interviews and reviewing the interview transcripts generated in Microsoft Teams, the team used a qualitative content analysis approach, hand-coded transcripts and documented additional barriers, clarifications to the system map, and new implementation strategies. Similar to the matrix analysis, the team did not seek information saturation given the small sample size of various healthcare professionals. They integrated the new content into the preliminary systems map.

All study members reviewed the transcripts for clarity, completeness, and consistency, documented barriers to early detection of CA, and ensured that the implementation strategies identified in the interviews were appropriately categorized and added to the map. Additionally, they ensured that each implementation strategy was documented and described. We applied these recommendations to the Expert Recommendations for Implementation Chance (ERIC) implementation strategies to further contextualize the strategies (14).

## 3 Results

Sixteen individuals (14 clinicians and 2 patients) were invited to participate in the workshop; however, 9 clinicians and 2 patients (*n* = 11 total) participated (68.8% response rate). Of the 14 clinicians invited for a follow-up interview, 10 (71.4% response rate) completed these member-checking interviews. See Table 1 for their details. Providers described numerous barriers to routinely conducting a diagnostic work-up for CA. Table 2 presents influences to timely work-up for CA that informed the expansion of the system map of care for CA. Participants also recommended implementation strategies to increase uptake of this evidence-based diagnostic protocol of bone scintigraphy and monoclonal protein testing for the early detection of CA. See Table 3 for the recommended strategies.

**Table 2:** Influences on the timely work-up for ATTR-CA.

System map category	Subcategory	Supporting quotes
Patients’ individual factors	Health literacy	Many patients have low health literacy. There needs to be a concerted effort to teach people a little about what it [cardiac amyloidosis] is. (Cardiologist 1)
	Genetic risk awareness	This could be a model for other diseases if we can convince people with this genetic disease to get genetically tested (Cardiologist 1)
Family	Hereditary	The V121I [genetic variant for ATTR amyloidosis] is a lot more common among African Americans. This is a genetic variant. Just the name itself would be useful to let them know. So, some simple message like “This is what we need you to know,” and then tell them all the symptoms. (Cardiologist 4)
	Advocacy	Family member advocacy is so important. If one family member has the diagnosis, they’re really the one who needs to share that information. (Genetic counselor)
Comprehensive history & physical examination	Physical examination	Part of going through the algorithm, I think, is a comprehensive history and physical examination, along with specific testing that focuses on the patient’s clinical presentation. (Primary care physician)
Initial clinical presentations	Heart failure	There must be clinical suspicion. I’ts a combination of cardiac and red flags, not just a single red flag… I’ts very rarely a single red flag. (Cardiologist 2)
	Bilateral carpal tunnel Spinal stenosis Bicep tendon rupture Neuropathy	You already have neuropathy, which is great. You have the big three with carpal tunnel, spinal stenosis, and biceps tendon rupture, which many people are now sending if they have surgery for carpal tunnel or spinal stenosis, sending those specimens to look for amyloid and then typing it … (Pathologist) And then if you come across this right atrial fibrillation, carpal tunnel syndrome, spinal stenosis, bicep tendon rupture, but if they also have neuropathy, please, please, please consider amyloid. (Primary care physician)
Additional screening	EKG/ECG (electrocardiogram)	You know, after they get an echo[cardiogram], someone hopefully tells them, “My suspicion is low or high.” (Cardiologist 1) EKG, of course, you know, before jumping into an ECHO, I think the EKG is always a really good tool, and then take it from there. Of course, the ECHO is going to be necessary– (Primary care physician) So, you have the ECHO with LVH, deceptive hypertrophy, the Mickey Mouse heart we all learn in medical school, and you have the EKG showing low voltage, but then you have LPH in the echo. I think the clinicians should see that as the biggest red flag. Cardiologist 1)
	Echocardiogram	After the primary care physician or the cardiologist met with the patient to discuss the symptoms and so forth, the patient would be scheduled for an echocardiogram. So, right at the beginning is the perfect place for that [in reference to the preliminary system map]. (Pathologist)
	MRI	The flow chart makes sense, and some things can change. For example, where does the cardiac MRI fit in? That should be included. (Cardiologist 1)
	Endomyocardial biopsy	So, depending on the case, for us to get confirmation, yes, we may say there is a high suspicion based on cardiac imaging. But if the test results are inconclusive or our suspicion remains high despite negative results, an endomyocardial biopsy would be helpful. Sometimes, if this is amyloid, we may say, “Well, let’s start with a fat pad biopsy or something less invasive.” But a biopsy can be helpful, especially when imaging results are inconclusive, and we don’t want to commit to treatment. (Cardiologist 3)
PYP + monoclonal protein		It’s going to lead us to believe you have a grossly abnormal aspect-free light chain, then get a hematologist on board—(Primary care physician)
Diagnosis		We need to add “diagnosis,” which is missing [from the map]. (Cardiologist 3)
System-wide barrier	Navigating the healthcare system	Because of the number of providers that they [patients] will see, they’re going to get lost in their system. You have to navigate them. (Cardiologist 4)

**Table 3:** Participants’ direct quotations about potential strategies to increase the early diagnosis of ATTR-CA.

Type of Strategy	More specific information about the strategy	Participant quote that supports the strategy
Marketing, Advertising Patient Education	Marketing	But this physician himself, who does primary care in the community, said he’s now been seeing these TV commercials for amyloid. And as a primary care physician, he’s like, “I’ve never cared for amyloid.” And I said, “You have, you just haven’t realized it yet.” (Cardiologist 2)
	Advertising	For the patient, raise awareness with the patient and their family. There could be marketing tools you could use. Advertising paired with this is where the pharmaceutical companies could also help further raise awareness that could prompt a patient to think, “Oh man, I’ve got these symptoms. It could be amyloidosis” (Primary care physician)
	Patient education	You need culturally sensitive information provided at each step. It is an opportunity for dialogue and conversation. (Cardiologist 1) There is an opportunity to simplify messaging for patients and engage in community health education, perhaps through a faith-based intervention in churches, healthcare settings, and even the pharmacy. (Cardiologist 4)
To facilitate cascade testing, leverage videoconference software to conduct family meeting to discuss hATTR risk	Test the proband’s first-degree relatives	We need to leverage the proband. We need to leverage the patient’s trust. We could create a Zoom meeting like we’re doing now and invite the proband and all family members. We could set up a one-hour meeting and tell them what is going on [patient’s diagnosis of amyloidosis]. We can send them all kits to get them tested. (Cardiologist 1)
Genetic counselors share genetic risk information	Letter to patient families	When I see someone who has a positive genetic test result, we find out that they have hereditary type amyloidosis. I write a family letter for that patient to give to their family members, and it really is just a basic one-page document that says, “Here’s what amyloidosis is. Here’s the gene that was identified or the gene variant that was identified in your affected family member. Here are resources for how you can get tested.” (Genetic counselor)
	Genetic counseling	Testing should be performed alongside genetic counseling. I give tips on how you can talk to a genetic counselor. Then, I include the website for locating a genetic counselor. (Genetic counselor)
Provider education	Various forms of provider education	So, if there’s an educational video for primary care physicians, maybe an educators’ forum for sonographers where those who are local or will be part of this can be trained on the specifics…(Cardiac sonographer) We give webinars to primary care providers to make people aware of it [cardiac amyloidosis]. (Cardiologist 1) So if there is a way to provide education to PCPs on what they should be looking out for… those earlier signs, definitely the screening, the hereditary kind of conditions that could sometimes increase one’s risk of amyloid or even screening more extensively a family history of if there is a strong family history of amyloidosis, all those things I think would go a really long way. (Cardiologist 3) It’s less challenging to target residents and their training, and to go as far as the ACGME [Accreditation Council for Graduate Medical Education]. And I would say that’s where societies and pharma can help, because then they could really drive home the messages—conversations about amyloid, its role in the cardiovascular system, and what that would look like beyond that. (Cardiologist 3)
Clinical decision support tools	AI + EKG	Dr. [X] and her team here at [healthcare system] have created an AI program that lets you take an EKG and run it through it, and it’ll give you a score indicating the patient’s risk of having amyloid. And it’s pretty darn good. (Pathologist)
	Increase clinical suspicion	Put this on your tool bucket list. Ask the question, “Could this be amyloid?” for every patient you see with heart failure. I think it should just be, “Could this be amyloid?” specifically for every heart failure patient with a wall thickness over 12. Now the US guidelines say 14. (Cardiologist 2)
	Use AI with electrocardiograms or echocardiograms.	So even doing a registry and then using artificial intelligence, you know, to identify patterns in EKG and ECHO that would raise that level of awareness, and in the differential diagnosis… bold underline and fluorescent colors. Tell the clinician you could be dealing with a case of cardiac amyloidosis. Go back, talk to this patient, and look at the past medical history. (Primary care physician)
	Use AI with electronic health records.	[Name] highlighted this morning, like the neuropathy in the absence of diabetes, the AFib in the absence of hypertension, certain things like that. So, leveraging AI and EMR to help guide physicians in making it somewhat dummy proof. (Cardiologist 2)
Tissue biopsy and routine stain	Stain tissues with Congo Red.	From the pathologist’s standpoint, but carpal tunnel is a good one. It’s not hard to do. It’s just a matter of getting it, getting it sent to pathology, and somebody remembering to do the Congo red well. (Pathologist)
	Ensure proper interpretation of nuclear imaging.	Not everyone in the Valley is using SPECT, and people still aren’t reading it properly, so they are not calling out the blood pool. Also, there can be false positives. [Name] from [name of institution] has talked about whether patients on Plaquenil and other things can have false positives. (Cardiologist 2)
Train sonographers to recognize amyloid	Educating sonographers to recognize amyloid	My suggestion was that there needed to be more education for the sonographers performing the screenings, because I don’t feel as if all of them truly understand how to recognize whether this patient could be a potential amyloidosis candidate. (Cardiac sonographer)
Patient takeback		Have patients who were misdiagnosed write some of their original providers nicely, non-litigiously, and tell them their story and say, “Hey. This is what happened to me. I went through X, Y, or Z. You saw me three years ago. And I just wanted you to know, so that maybe you’ll think about this disease in the future.” (Cardiologist 2)
Patient navigation		For [patient] navigation, was there an intention to have a patient navigator walk them through the diagnostic process? Because that would be ideal. (Cardiologist 3)
Patient advocacy groups		This is a very tight-knit community. They’re all involved in the Facebook groups, and ASG [Amyloidosis Support Group] is probably spearheading this. Then their information for all of these different things is disseminated through ASG and the ASG website…So, through word of mouth, through these patient networks and support groups, they’re all learning from one another. (Cardiologist 2)
Provider team specializing in CA	Team approach to diagnosing and treating amyloid	Having a dedicated group of people who understand amyloid across these different specialties… that’s a great place to start. (Genetic counselor)
Provider directory to find amyloid specialists	Create a registry of providers who specialize in cardiac amyloidosis.	The registry… I think it would be helpful to provide information on who to reach out to, such as some of the cardiology program directors, and to see what they think. We can talk to Doctor X [name] at Y [healthcare system]. Can we talk to Dr. X [another provider name], who finished a fellowship training in cardiac imaging? (Primary care physician)

**Figure 1:**
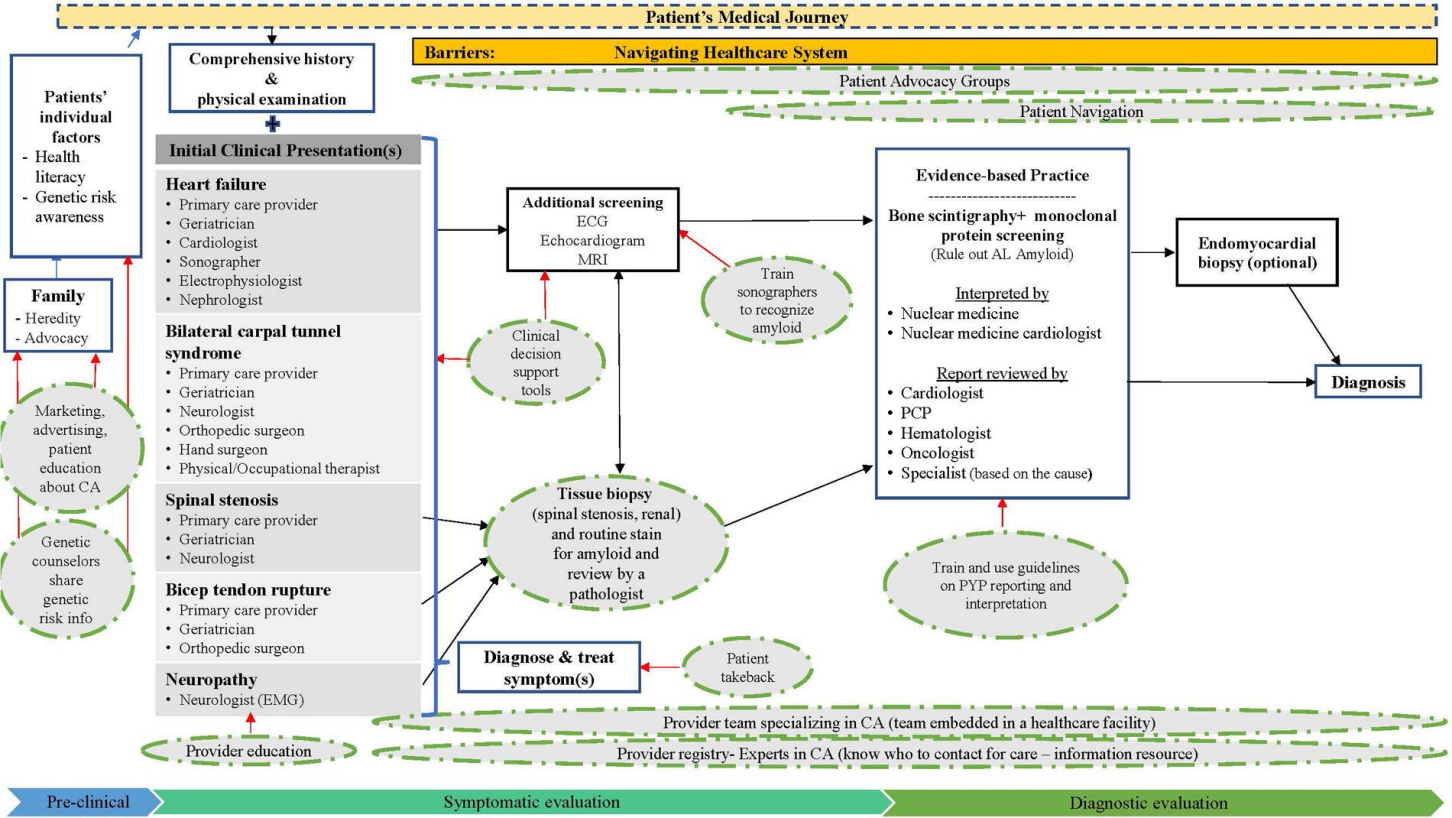
System map of care with implementation strategies to enhance the early detection of cardiac amyloidosis. Reproduced from “Revised system map for the early detection of CA” by Sherry L. Ball, Alexis Koskan, Jenice Guzman and Sandesh Dev, CC0 1.0.

The research team organized the updated map into three phases of the patient care experience for CA: pre-clinical, symptomatic, and diagnostic evaluation. Implementation strategies are broadly grouped (e.g., clinical decision support tools and provider education). Figure 1 illustrates the final systems map for CA. In the map, the black arrows illustrate patients’ steps in receiving CA-related care; green dashed circles are the implementation strategies identified through this research; red arrows illustrate the implementation strategies' potential impact on the patient experience, and the blue arrow illustrates how a patient's individual factors may influence their medical journey. Table 4 provides a more detailed description of the individual implementation strategies that can help influence the eventual uptake of bone scintigraphy and monoclonal protein screening.

**Table 4:** Implementation strategies to increase the early detection of CA.

IS #	Categorized in CA early detection system map of care	Who receives strategy	Implementation strategy and description	ERIC strategy
Pre-clinical stage testing				
1	Marketing, advertising, and patient education	Patient	Develop easy-to-understand, plain-language information about CA	Develop educational materials
2	Marketing, advertising, and patient education	Patient	Use mass media to market and advertise information about CA to the public	Use mass media
3	Marketing, advertising, and patient education	Patient	Distribute CA educational materials where populations at risk (including older adults) are more likely to see the information (e.g., outpatient pharmacies, retirement communities)	Develop educational materials
4	Share genetic risk information	Patient	Involve family members with h-ATTR to inform their first-degree family members of their increased risk for CA	Involve patients/consumers and family members
5	Share genetic risk information	Patients’ first-degree relatives	Communication about genetic risk for CA	Genetic counselors share information with first-degree relatives of patients with h-ATTR to increase their risk awareness for the disease and prompt genetic testing and counseling for CA
Symptomatic evaluation				
6	Clinician education	Clinician	Develop educational materials for clinicians to learn about and recognize the red-flag symptoms of CA	Develop educational materials
7	Patient take-back	Patient and Clinician	Patient take-back: Encourage patients previously misdiagnosed or underdiagnosed for CA to return to their healthcare clinicians who missed the diagnosis for CA to educate clinicians and potentially prevent reoccurrence with future patients	Involve patients/consumers and family members
8	Clinician education	Clinician	Offer multiple modalities to train clinicians, which include: Didactic curriculum paired with case studies of patients with CA; Academic half-day sessions about CA for medical students and fellows; Oral review about CA for medical residents; Shadowing opportunities for medical students and fellows to observe the diagnosis and treatment of a patient with CA; CME events focused solely on CA diagnosis and care; Trainings and fellowships sponsored by national organizations and pharmaceutical companies to help clinicians become experts in the early detection and treatment of CA; Case studies and videos to train sonographers to look for specific changes in the echocardiogram; Academic detailing: Medical liaison or consultant meets with practitioners to offer in-depth training on the detection and treatment of CA	Work with educational institutions; Develop educational materials, conduct educational meetings; Make training dynamic; Shadow other experts, Recruit, designate, and train for leadership; Conduct educational outreach visits; Capture and share local knowledge, Conduct ongoing training
9	Clinical decision support tools	Clinician	In-clinic clinician prompts (such as a poster) to consider amyloid as the underlying cause of a multi-symptom disease	Remind clinicians
10	Clinical decision support tools	Clinician	Create patient registries of heart failure (and other at-risk) patients and use AI to scan patient files to identify individuals at risk or who exhibit a likelihood of having CA	Use data warehousing techniques
11	Clinical decision support tools	Clinicians & Staff	Electronic health record prompts to consider amyloid	Change record systems; Remind clinicians
12	Clinical decision support tools	Clinicians & Staff	Explicitly document CA suspicion on a patient’s echocardiogram	Facilitate relay of clinical data to providers;
13	Clinical decision support tools	Clinician	Increase provider awareness to implement ATTR risk calculators in the electronic health record systems	Facilitate relay of clinical data to providers;
14	Clinical decision support tools	Clinician	Create an amyloid order set to refer patients to additional specialists for the early detection of CA (if CA is suspected)	Assess and redesign workflow, Change record systems
15	Tissue biopsy	Clinician	Routinely request Congo red staining for tissue biopsies	Assess and redesign workflow
16	Tissue biopsy	Clinician	Train pathologists to conduct Congo red staining	Conduct ongoing training
17	Train sonographers to recognize amyloid	Clinicians & Staff	Train sonographers to perform strain imaging	Conduct ongoing training
Diagnostic evaluation				
18	Train and use guidelines on PYP reporting and interpretation	Clinician	Train clinicians working in nuclear medicine to follow guidelines on bone scintigraphy reporting and interpretation	Provide clinical supervision
19	Train and use guidelines on PYP reporting and interpretation	Clinician	Conduct interrater reliability of bone scintigraphy imaging reports	Develop and organize quality monitoring systems
Multiple healthcare stages				
20	Patient navigation	Healthcare system	Patient navigation for CA—beginning as early as the additional screening and ending after the surveillance period	Intervene with patients/consumers to enhance uptake and adherence
21	Patient advocacy groups	Patient, Clinician, Staff	Encourage patients to work with patient advocacy groups	Involve patients/consumers and family members; Prepare patients/consumers to be active participants
22	Provider team specializing in CA	Healthcare system	Create teams of clinicians trained in identifying and treating CA	Create new clinical teams
23	Provider registry	Clinician	Create a clinician database of community CA experts	Identify early adopters, Develop resource sharing agreements

### 3.1 Pre-clinical stage: barriers and implementation strategies

Participants noted the lack of patient knowledge as a barrier naming characteristics in seeking care for CA symptoms, including general awareness of CA, knowledge of CA symptoms, health literacy, family history/genetic status [for those with family members with hereditary ATTR (hATTR)] and related risk perceptions for CA as impacting the likelihood that patients will act on concerning health information or facilitators to seeking care. Participants suggested multiple mechanisms to promote patient education as an implementation strategy to increase the number of patients with clinical risk factors who discuss testing for CA with their providers. (IS1 in Table 4). Educational materials could be disseminated via patient-directed advertising (including social media marketing), to help patients recognize CA-associated signs and symptoms and thereby increase the likelihood of seeking care (IS2). These materials can be distributed at places where older adults frequent and among communications they use [e.g., American Association of Retired Persons (AARP) newsletters]. Another recommendation was to have pharmacies distribute CA informational fliers to patients with heart failure, given their increased risk for the condition (IS3).

Participants noted that patients did not always share genetic risk information for ATTR with family members who may have inherited the same genetic mutation. For patients with hATTR, the lack of sharing genetic mutation and ATTR-CA risk information was a barrier for their first-degree relatives to seek early detection. Participants noted that patients with hATTR should be encouraged to share their genetic status with family members to facilitate cascade testing, testing all first-degree relatives to identify affected relatives (IS4). A suggested implementation strategy was to have genetic counselors working with hATTR patients draft a letter to patients' first-degree relatives (i.e., siblings and children) that the patient can then give them to encourage them to undergo genetic testing. Such a letter confirming the first-degree relative's genetic diagnosis can facilitate appropriate genetic test selection and provide documentation for health insurance companies to cover the costs of genetic testing (IS5).

### 3.2 Symptomatic evaluation: barriers and implementation strategies

Access to a complete patient history was noted as a barrier to reaching a diagnosis. Participants emphasized the importance of comprehensive history-taking with a physical examination to the symptomatic evaluation stage of care in the map, tasks often carried out by a patient's primary care provider. One participant emphasized the need to provide in-depth training on CA for clinicians who work with older adults, given the increased prevalence of CA in older adults (IS6). Clinicians treating non-cardiac “red flag” symptoms (e.g., bilateral carpal tunnel syndrome, spinal stenosis, bicep tendon rupture) may only diagnose and treat the symptoms rather than correctly identify the underlying disease. One participant recommended that patients with CA improve clinician awareness by meeting with clinicians who previously misdiagnosed their CA or treated their symptoms only to share their diagnosis in a strategy they called a “patient take-back” (IS7).

To overcome clinicians' lack of clinical suspicion for CA, participants recommended multiple methods for clinician training and education (e.g., offering CA-focused fellowships, hosting CME events, and presenting CA case studies to clinicians), outlined in Table 1 (IS8). Further, to prompt clinical suspicion of CA, participants recommended creating and posting clinician reminders, such as posters in clinical examination rooms, to prompt clinicians to consider amyloid as the cause of the patient's symptoms (IS9).

Participants discussed challenges and lack of experience in ordering and interpreting follow-up diagnostic tests, including electrocardiogram (EKG/ ECG), echocardiogram, and cardiac magnetic resonance imaging (MRI). Test results may indicate the need for additional CA testing. Barriers for clinicians interpreting these tests included recognizing characteristic features in the test results and noting the presence of other CA-related symptoms. In response to these challenges, participants described various clinical support tools to aid in the early detection of CA. Participants recommended creating patient registries that include clinical characteristics suggesting CA risk factors, such as electrocardiogram, echocardiogram, and cardiac MRI findings. These registries could utilize artificial intelligence (AI) to analyze patients' electronic health records (EHR) and imaging reports to identify at-risk patients (IS10). Such registries could be paired with alerts to prompt clinicians to order CA diagnostic tests (IS11). One cardiologist and CA expert recommended using clear language in imaging reports, such as explicitly writing “amyloid” in echocardiogram reports, to prompt clinician attention (IS12). The same clinician recommended using a clinical decision support tool to calculate the risk of a patient having ATTR-CA (IS13) (15). The clinician recommended its use to consider CA as a potential differential diagnosis. Another cardiologist described how her clinic had an “amyloid order set,” a comprehensive collection of medical assessments and referrals used to detect CA, which facilitated proper referral to specialists for diagnostic evaluation of CA (IS14).

The clinicians who treat the non-cardiac initial symptoms of CA could order tissue biopsies (e.g., spinal tissue from spinal stenosis, renal tissue from gastrointestinal disorders, tissue during carpal tunnel release) and routinely request Congo red staining for amyloid (IS15). Further, participants recommended that pathologists receive additional training to properly perform Congo red staining, as there are often errors in conducting the test and interpreting the results (IS16). Additionally, participants suggested that sonographers receive training in recognizing amyloid by incorporating strain speckle tracking into their echocardiographic workflow, which could improve recognition of the CA phenotype through characteristic strain patterns (IS17).

### 3.3 Diagnostic evaluation: barriers and implementation strategies

Participants described challenges, including a need for more specialized knowledge to carry out evidence-based practices in bone scintigraphy and monoclonal protein screening. Such challenges include the need for single-photon emission computed tomography (SPECT) (rather than only planar imaging), the need for standardized training (of nuclear medicine technologists) for bone scintigraphy procedures, and the need for better quality control for reporting and documenting the results. One participant noted the need for nuclear medicine-qualified physicians (including cardiologists) to interpret bone scintigraphy correctly. To overcome these challenges, participants recommended ensuring that all who interpret and review the scan results follow nuclear medicine guidelines, particularly the American Society of Nuclear Cardiology's Practice Points for ATTR-CA PYP imaging (IS18) (16). Additionally, one participant proposed a quality control process of measuring interrater reliability among those reviewing the scans, to ensure they (IS19).

### 3.4 Cross-cutting barriers and implementation strategies

Participants noted barriers to navigating a complex, fragmented healthcare system. At the healthcare system level, participants recommended that hospitals employ patient navigators, individuals who assist patients in navigating a complex healthcare system from CA early detection to diagnosis and treatment. (Roles of patient navigators often involve scheduling appointments, providing health education, following up with patients, identifying resources such as transportation or financial assistance, etc.) They noted that patient navigators help patients overcome gaps in timely care and follow-up (IS20). Additionally, they recommended linking at-risk individuals to patient advocacy groups, such as the amyloidosis support groups, to provide support for patients before and after receiving a CA diagnosis (IS21).

Participants described a need for multispecialty teams organized around caring for patients with CA. Another participant noted the need for clinicians to work in “medical villages” rather than “medical silos,” and highlighted the difficulty of making a diagnosis without coordinated patient information from the multiple providers who may be treating CA symptoms. In response, some participants recommended creating “amyloid teams,” assembling dedicated clinicians with additional training to screen for, identify, and treat CA (IS22). Participants also described uncertainty regarding where to refer patients they suspect have CA in their local community. They discussed creating a database of local clinicians who specialize in treating this disease (IS23).

## 4 Discussion

This research focuses on identifying distinct, interlinked implementation strategies spanning multiple phases of the patient care journey and carried out by various specialists in a complex, adaptive system. It moves beyond describing diagnostic algorithms that that lead to the early detection for CA (17). However, it is critical to note that observing such algorithms and implementing clinical pathways can lead to clinicians' increased recognition of ATTR-CA red flags (18). Other researchers found that implementing clinical pathways increased diagnosis for CA with reduced diagnostic delay and reduced CA severity at time of patients' diagnosis (19). By implementing and testing the strategies identified in the current research and using a systems science lens, patients are expected to receive more timely evaluation for ATTR-CA by completing the evidence-based diagnostic tests. This study highlights implementation strategies often employed in quality improvement initiatives, such as providing clinicians with appropriate education (20), clinical support tools (21), and coordinated care (22). It also validates CA-specific strategies, such as encouraging those at risk for hATTR to undergo genetic testing for an ATTR gene variant (23). Additionally, we identified new, unexpected implementation strategies, including patient “take-back,” conducting biopsies during orthopedic surgeries, training sonographers to recognize amyloid, and developing recommendations to improve the quality of nuclear scintigraphy imaging procedures and documentation.

### 4.1 Common implementation strategies

Concordant with past research, participants reported underdiagnosis or late diagnosis of CA due to a lack of recognition and a lack of clinical suspicion. For example, one study estimated that patients were diagnosed with CA two years after experiencing multiple (non-specific) symptoms and after seeing a minimum of five physicians (24). To prompt clinicians to consider CA, our participants described numerous educational strategies. One novel strategy is patient “take-back,” in which a CA patient with a delayed diagnosis would share their diagnostic experience with their original clinician, either in person or by letter. This strategy may empower patients and allow their clinicians to learn from them. This strategy appears unique because it is not described in the literature, and it supports other efforts to collect patient-reported experiences to reduce diagnostic errors in health systems (25).

Participants recommended clinical decision support tools to help clinicians detect CA early. Some participants recommended using new technologies and AI paired with patient registries to screen for CA. Such tools that utilize clinical characteristics (26), electrocardiogram (27), echocardiogram (28), and MRI data (29) are reported in the literature. Past research also reports on the utility of AI and machine learning techniques to examine electrocardiogram reports among patients with severe aortic stenosis (pre-transcatheter aortic valve replacement) to detect patients at higher risk for negative clinical outcomes with CA (30). However, a more recent study recommended using machine learning techniques with CT strain data to identify ATTR-CA among patients with severe aortic stenosis (31). Current research is exploring the clinical value of using AI along the disease spectrum (32).

Due to these varied presentations and the complexity of the disease, a recommended strategy was to create multidisciplinary amyloidosis care teams, which can more holistically and effectively guide a patient with CA throughout the entire patient care journey. Such teams have been employed in healthcare systems (e.g., Centers of Excellence), similar to the “heart team” required for hospitals performing transcatheter aortic valve replacement (TAVR) (33, 34). The recommendation to form such expert teams in CA highlights the need for established standards or consensus for what qualifies as a “qualified amyloid team.” We do not believe such a standard currently exists, although some researchers have proposed criteria (35). For healthcare clinicians not working with such expert teams, ta directory of clinicians in their community who treat amyloid can help clinicians identify other professionals with CA expertise who can effectively treat and manage patients with CA.

### 4.2 New implementation strategies

Although amyloid guidelines recommend screening all first-degree relatives of patients with hATTR (36) approximately 10 years before the proband was either symptomatic or diagnosed with ATTR-CA (37), we did not anticipate the importance of the patient and family factors (e.g., trust in genetic testing and willingness to undergo genetic testing) or the suggested communication strategy (e.g., test patients with CA for genetic mutations at the time of diagnosis and communicate outcomes to family members immediately) to improve the implementation of cascade testing (38). In one multi-center study, family members carrying the genetic variants who participated in cascade genetic screening had a 57% lower risk of mortality compared to probands (39). It is critical that first-degree relatives of the index patient (proband) undergo genetic testing for hATTR, especially because this form of CA affects younger patients (e.g., median age of diagnosis is 39 years) than other forms of CA (40). Additionally, the European Society of Cardiology Working Group on Myocardial & Pericardial Diseases recommended family members who test positive for the ATTR-CA genetic variants, but who are asymptomatic should continue screening for ATTR-CA via bone scintigraphy every three years to detect this condition at its earlier stages (41). Research evaluated these recommendations, and found that, in clinical practice, they performed well (42). We encourage future research to examine best practices and strategies (e.g., patient letters, emails, in-person communication, and clinician phone calls) for communicating CA genetic risk to family members and encouraging their genetic testing and counseling.

Clinicians recommended strategies directed at pathological diagnosis. One recommended strategy was to routinely perform tissue biopsies in patients undergoing surgery for CA “red flags” such as carpal tunnel syndrome and spinal stenosis (43). Although this strategy has been reported in the case of carpal tunnel release surgery, it is not yet the standard of care (44). If validated, then these strategies could represent a paradigm shift in the screening of pre-symptomatic individuals with carpal tunnel syndrome or spinal stenosis and require significant coordination among pathologists, surgical specialists, neurologists, and cardiologists. Another related and unanticipated strategy is to train pathologists to perform Congo red staining correctly, a vital recommendation given that Congo red staining is the initial test for pathologic samples suspected of amyloid (45). Clinicians must understand the limitations of this test (46) and perform it as accurately as possible. This potential performance gap underscores the importance of a systems approach in defining barriers to optimal amyloid diagnosis.

Regarding the quality of echocardiography, the recommendation to train sonographers in strain imaging underscores the importance of allied health professionals within the broader system of care supporting CA diagnosis. This recommendation is important because there is a relative dearth of research focusing on sonography education (47).

Study participants described the importance of high-quality bone scintigraphy and echocardiography imaging and the risk of misdiagnosis if performed incorrectly. Their recommendations for bone scintigraphy were consistent with professional society recommendations that emphasize the need for SPECT over planar imaging and a 2- to 3-hour time interval to avoid excess blood-pool activity (48). The importance of proficiency in bone scintigraphy imaging and interpretation is also linked to the strategy of clinician education. Another recommended strategy was to employ a double reading of bone scintigraphy results (49), in which two radiologists read the same results to improve accuracy. This is a potentially valuable strategy because the interobserver reproducibility of bone scintigraphy studies has not been well studied beyond high-volume amyloid centers (50).

As is evident from both Figure 1 and subject matter experts' repeated emphasis on a “team” and non-siloed approach, successful CA early detection and diagnosis depend on several healthcare system components working together, not on cardiology alone. Efforts at implementation should employ a systems science approach, using multi-level strategies that span multiple stages of a patient's journey (51). This approach contrasts with a reductionist, siloed approach, where workflows and individual implementation strategies are tested independently, ignoring the relationship between upstream and downstream components.

### 4.3 Limitations and considerations

First, the research team included clinicians who participated in both the design and data collection for this study. This may impact the interpretation of study results, compromising the internal validity of study findings. Another limitation of this study is the use of a convenient, purposive sample of patients and clinicians. However, this is appropriate for qualitative research that relies on expert feedback to explore challenges in healthcare delivery. Another limitation is that the healthcare professionals who participated in the study worked across different healthcare institutions and settings, which limits the internal validity of this system map but likely enhances the external validity of our findings. Some clinician participants worked in hospital systems with protocols and teams designated for caring for patients with CA. In contrast, others worked in community hospitals and clinics that treat CA patients but lack the same infrastructure and frequency of CA treatment as the Centers of Excellence. We believe that having providers representing a variety of healthcare settings is a unique strength of this study, as expert panels are often limited to tertiary or quaternary care centers. However, future research should consider incorporating additional perspectives from health system administrators, pharmaceutical scientists, and medical trainees, whose influence on referral logistics, prior authorization processes, and out-of-pocket expenses profoundly shapes the real-world feasibility of any proposed intervention that stems from this work. Another participant-related limitation is that all included patients were identified by a single expert cardiologist, which risks selection bias, as patients chosen by an expert at an amyloid Center of Excellence may not represent the broader population of delayed-diagnosis patients.

## 5 Conclusion

Despite the availability of evidence-based practice guidelines on diagnosing CA, this condition is often undetected, leading to excess morbidity and mortality. Barriers to CA diagnostic testing broadly relate to an uncoordinated, fragmented, complicated healthcare system and a non-specific multisystem disease, of which red flag symptoms mirror less fatal causes. Our research, one of the earliest efforts to map barriers and facilitators to CA diagnosis, identified 23 distinct implementation strategies that can be evaluated to enhance CA early detection through the evidence-based practice of bone scintigraphy with monoclonal protein testing. This area of medicine and research calls for implementation scientists to conduct multilevel interventions involving various clinicians, including, but not limited to, those working in cardiology, primary care, neurology, imaging, surgery, and genetic testing and counseling. Future research or implementation efforts should tailor the CA system map to a specific healthcare system before selecting implementation strategies and creating an implementation blueprint to increase the evidence-based CA early-detection strategy of bone scintigraphy with monoclonal protein testing.

## Data Availability

The raw data supporting the conclusions of this article will be made available by the authors, without undue reservation.

## Appendix 1 Follow-up interview Guide

**Thank you for taking the time to evaluate the preliminary systems map for the early detection of cardiac amyloidosis. We took information from the workshop we conducted in January 2023 to create this preliminary map. We want to get your feedback on how we can improve this map.**

- What is missing from this map?
- What/how would you add/change to this map?  
  - Who else plays a role?
- Is there anything else that should be on this map that may happen earlier in time or outside the space we have on this map?
- Where are there opportunities for improvement?
- Are there any other strategies you recommend to promote the timely screening for CA?

**Thank you all for your time. We will update this map according to your feedback.**
